# Blood Ordering and Transfusion Practices: An Insight Toward Better Utility of Blood Products

**DOI:** 10.7759/cureus.22075

**Published:** 2022-02-10

**Authors:** Samra Waheed, Munira Borhany, Madiha Abid, Imran Naseer, Tahir Shamsi

**Affiliations:** 1 Hematology, National Institute of Blood Diseases and Bone Marrow Transplantation, Karachi, PAK

**Keywords:** crossmatch, random donor platelets, blood transfusion, transfusion index, transfusion probability, transfusion ratio

## Abstract

Background

An adequate supply of quality blood products is the backbone of any hospital. To maintain it, the utilization and wastage of the products should be closely monitored.

Objective

To determine the crossmatch to transfusion (C/T) ratio, transfusion probability (%T), and transfusion index (Ti) of packed red blood cells and to review the use of platelets.

Materials and methods

A total of 6,326 hematological patients receiving packed red blood cells were included in the study. The random donor platelets that were prepared during this period were also included to know the actual utilization of platelets.

Results

A total of 26,146 crossmatches were requested for these 6,326 patients in three years. Out of these, 26,024 units were issued and transfused to the patients. The CT ratio of our data was calculated to be 1.00, the transfusion probability was found to be 98.1%, and the transfusion index was computed to be 0.99. For random donor platelets, 37,162 were prepared from whole blood during this period, while 30,971 platelets were transfused to the patients.

Conclusion

The overall results of our analysis showed proper utilization of blood products at our institution. The wastage was considered to be minimal.

## Introduction

In a clinical laboratory, the blood transfusion services (BTS) occupy an exclusive slot as it holds three components: one concerned with the collection of blood products; the second devoted to the preparation, allocation, and diagnostics of blood banking; and the third being the clinical and remedial constituent. The constitutional services of a blood bank are primarily therapeutic rather than diagnostic, which is in contrast to other pathology subspecialties [1]. Impulsive over-ordering of blood can strain the physical and the human budget of a healthcare system and therefore increase the overall expenditure of patient care, especially in resource-constraint settings. To reduce the complexities and to work with minimal wastage of resources, a multidisciplinary approach is required in the utilization management of blood products.

The need for blood products in hospitals is constantly increasing, which is reflected in the volume collected by the transfusion services throughout the globe. During the coronavirus pandemic, specifically, it has become awfully difficult to meet the transfusion requirements, especially in developing countries, due to an increased number of consanguineous marriages. The common autosomal recessive disorders following consanguineous marriages include thalassemia, bleeding disorders, and bone marrow failure. Studies have shown that there is often a massive over-ordering of blood in nonsurgical and surgical settings [2]. Moreover, because of the over-ordering of blood by clinicians, the nonavailability of the crossmatched units remains a significant issue as they are kept for specific patients whose transfusion requirements are uncertain [3]. All the abovementioned reasons inflict storage issues for the blood bank, loss of shelf life, and wastage of the product [4].

A number of indices can be used in the transfusion services to persuade the efficacy of blood ordering and utilization system. Boral Henry in 1975 first recommended the use of crossmatch to transfusion (C/T) ratio [5]. Thereupon, numerous authors, especially in surgical patients, used the C/T ratio to evaluate blood transfusion practice. Precisely, this ratio should be 1.0, but a ratio of 2.5 and below was advocated to be demonstrative of efficient blood usage [6].

Reviewing blood ordering and transfusion practices along with enrooting of a blood ordering program can provide predictable blood usage in surgical and nonsurgical patients and thus help in reducing blood wastage, human resources, and most importantly financial burden on the patients [7].

The principal aim of our audit is to evaluate, review, and improve the efficiency of the current transfusion practices in hematology patients using different blood utilization indices.

## Materials and methods

We conducted this retrospective analysis at the National Institute of Blood Diseases and Bone Marrow Transplantation from January 2017 to December 2019. A total of 6,326 hematological patients were included in the study. Patients requiring any surgery were excluded. Informed consent was taken from blood donors. The following three indices were calculated for each patient separately: crossmatch to transfusion (C/T) ratio, transfusion probability (%T), and transfusion index (Ti) (Table 1).

**Table 1:** Utilization of blood indices Data of random donor platelets prepared during this time period were also gathered.

Crossmatch to transfusion (C/T) ratio	Transfusion probability (%T)	Transfusion index (Ti)
Number of units crossmatched / number of units transfused	Number of patients transfused / number of patients crossmatched × 100	Number of units transfused / number of patients crossmatched
A ratio of 2.5 or below is considered significant for blood usage. The probability of a transfusion (%T) for a given procedure was suggested by Mead et al. and others [8,9]. A value of 50% and above has been suggested as appropriate [3].	A value of 30 or more was suggestive of significant blood usage. Transfusion index (Ti) signifies the appropriateness of the numbers of units crossmatched. A value of 0.5 or more is indicative of efficient blood usage [3,5].	A value of 0.5 or more was demonstrative of significant blood utilization [3].

The use of crossmatch to transfusion (C/T) ratio was first recommended by Boral Henry in 1975 [5]. Ideally, this ratio should be 1.0, but a ratio of 2.5 and below was suggested to be indicative of efficient blood usage [3].

Statistical analysis

The collected data was entered and analyzed using the SPSS software version 23 (IBM Corporation, Armonk, NY, USA). Relevant descriptive statistics such as frequencies, percentages, and previously mentioned indices were calculated from the data. Finally, the results of the study were presented using narration statements and tables.

## Results

A total of 6,326 patients were recruited in the study, with 3,367 males and 2,959 females, with the mean age being 23.7 ± 22.3 years. The demographic characteristics of the patients are presented in Table 2.

**Table 2:** Demographic characteristics of the patients

Number of patients	6,326
Age (mean ± SD)	23.7 ± 22.3
Male (%)	3,367 (53.25)
Female (%)	2,959 (46.7)
Number of crossmatched	26,146
Number of units transfused	26,024
Number of wastage	122

**Figure 1:**
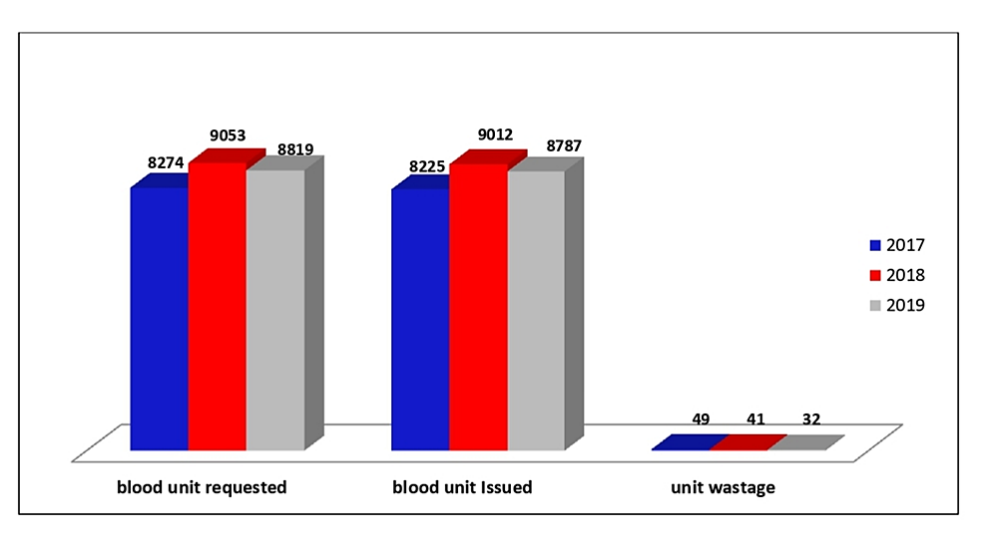
Yearly distribution of packed red blood cells crossmatched

A total of 26,146 crossmatches were requested for these patients in three years. Figure 1 illustrates the yearly distribution of packed red blood cells crossmatched and issued in the blood bank.

Out of these, 26,024 units were issued and transfused to the patients. A total of 108 crossmatched units were not issued from the blood bank, and 14 of the units were issued, but not transfused, to the patients on clinical grounds. Hence, these 122 units were not transfused to the patients and were considered to be a part of transfusion wastage. A total of 114 patients did not receive these crossmatched units. Overall, 673 irradiated red cells were transfused to the patients, whereas there were 543 washed packed red blood cells. All the washed and irradiated packed red blood cells were transfused to the patients. The CT ratio of our data was calculated to be 1.00, the transfusion probability was found to be 98.1%, and the transfusion index was computed to be 0.99, as demonstrated in Table 3.

**Table 3:** Overall indices of transfusion units

Crossmatch to transfusion (C/T) ratio	1.0 ratio
Transfusion probability (%T)	98.1%
Transfusion index (Ti)	0.99

As for platelets, 37,162 random donor platelets were prepared from whole blood from 2017 to 2019. Out of these, 31,345 platelets were allotted to the patients. The remaining 5,817 platelets were wasted either due to the expiry of the product or due to the positive screening of the donor. Table 4 shows the yearly distribution of platelets used.

**Table 4:** Utilization and wastage of random donor platelets

Product detail	2017	2018	2019	Total
Total prepared	10,825	14,915	11,422	37,162
Allotted	9,664	12,395	9,286	31,345 (84.3%)
Issued	9,560	12,222	9,189	30,971 (83.3%)
Discarded	580	1,847	1,689	4,116 (11.0%)
Reactive	685	846	544	2,075 (5.5%)

## Discussion

Blood is a precious clinical resource that should not be wasted. In the majority of hospitals, especially the surgical units, huge numbers of blood units are crossmatched each day for patients who rarely require transfusion, which results in the loss of resources [8,9]. The optimal functioning of a blood bank is aided by an efficient availability of blood products. According to the recommended value of CT ratio (<2.5 or ideally 1.0), our study demonstrates efficient blood usage as our CT ratio has been measured as 1.0. This finding correlates with some of the studies representing nonsurgical patients and minimal blood wastage [10], and it also coincides with a study from Iran [11], where type and screen (T&S) policy or electronic crossmatch has been used. Despite our study using a conventional crossmatching method, our hospital managed to minimize the loss of resources. Many other studies contradict our findings [12-14] because of a high CT ratio, specifically in surgical patients and those coming to the emergency department, leading to the insufficiency of blood utilization and increased wastage of blood, finances, and human resources. In our study, only patients with hematological disorders were included because the majority of these patients require transfusions most of the time. To the best of our knowledge, this is the first study analyzing patient blood management in hematological disorders. Few studies from New Zealand and Australia represent a CT ratio of 1.3 even in ICU patients, which demonstrates that these countries follow strict transfusion guidelines [15].

Moreover, the transfusion probability in our study was 98.1%, which was also consistent with the significant blood usage and is in accordance with a study performed in Pakistan [10]. However, another study from Pakistan showed a transfusion probability of 7.1% in surgical patients [12]; these findings are in line with the fact that maximum blood wastage is considered to be from surgical wards worldwide.

Furthermore, the last component of our study was to calculate the transfusion index (Ti), which was estimated to be 0.99 and also signifies valid blood usage. These findings also correspond to one study where the Ti was 0.99 in both surgical and nonsurgical patients [10]. In another study, the Ti was suggestive of excessive blood wastage (0.09) in patients with elective surgical procedures [12].

The utilization of random donor platelets was more than 80% in our study, which indicates optimum consumption of platelets in our institution since there are no specific guidelines for platelet transfusion in Pakistan. The remainder 11% of the platelets were wasted due to the expiry of the products, and 5% of the platelets were discarded because of positive screening of the donors. In a study done in India, platelet utilization was reported to be 43% [16], whereas the utilization of random donor platelets at our institution was better as all of our patients were related to hematological disorders, thus requiring more platelet transfusion.

A drawback of our study is that we excluded surgical patients; hence, we could not compare the two groups. Another limitation is that we did not subdivide patients according to their diagnoses so we could assess the possibility of product wastage conferring with the diagnosis.

## Conclusions

The practice of an appropriate blood ordering schedule can significantly reduce the expenditure of the patient and manage the blood bank inventory in a better way, thus making the blood products available to those who actually need them. Every hospital should have a blood transfusion committee that makes policies for blood utilization and wastage and conduct regular audits. On the basis of our findings, we can conclude that our hospital is fulfilling the requirements of blood ordering and transfusion practices.

## References

[1] Peña JR, Dzik WS (2014). Utilization management in the blood transfusion service. Clin Chim Acta.

[2] Vibhute M, Kamath SK, Shetty A (2000). Blood utilisation in elective general surgery cases: requirements, ordering and transfusion practices. J Postgrad Med.

[3] Olawumi HO, Bolaji BO (2006). Blood utilization in elective surgical procedures in Ilorin. TJHS.

[4] Jayaranee S, Prathiba R, Vasanthi N, Lopez CG (2002). An analysis of blood utilization for elective surgery in a tertiary medical centre in Malaysia. Malays J Pathol.

[5] Friedman BA, Oberman HA, Chadwick AR, Kingdon KI (1976). The maximum surgical blood order schedule and surgical blood use in the United States. Transfusion.

[6] National Health Service (1998). Health Service Circular HSC 1998/139. https://scholar.google.com/scholar?hl=en&as_sdt=0%2C5&q=Executive+NH.+Health+Service+Circular+HSC+1998%2F139.&btnG=.

[7] Silberstein LE, Kruskall MS, Stehling LC (1989). Strategies for the review of transfusion practices. JAMA.

[8] Lowery TA, Clark JA (1989). Successful implementation of maximum surgical blood order schedule. J Med Assoc Ga.

[9] Mead JH, Anthony CD, Sattler M (1980). Hemotherapy in elective surgery: an incidence report, review of the literature, and alternatives for guideline appraisal. Am J Clin Pathol.

[10] Rehan M (2016). Blood cross-match ordering practices. J Rawalpindi Med Coll.

[11] Alavi-Moghaddam M, Bardeh M, Alimohammadi H, Emami H, Hosseini-Zijoud SM (2014). Blood transfusion practice before and after implementation of type and screen protocol in emergency department of a university affiliated hospital in Iran. Emerg Med Int.

[12] Soomro R, Javed MR, Ali SA (2018). Blood transfusion: arrangements and use of blood in elective surgical procedures. Prof Med J.

[13] Chawla T, Kakepoto GN, Khan MA (2001). An audit of blood cross-match ordering practices at the Aga Khan University Hospital: first step towards a maximum surgical blood ordering schedule. J Pak Med Assoc.

[14] Baraka A, Juma T, Asfar SK, al-Sayer H (1991). Conserving blood in preparation for elective surgery. J R Soc Med.

[15] Westbrook A, Pettilä V, Nichol A (2010). Transfusion practice and guidelines in Australian and New Zealand intensive care units. Intensive Care Med.

[16] Bansal K, Kakkar R (2017). Study of the ratio of cross-matching to transfusion of blood or blood component, i.e. packed red blood corpuscles to develop good practices for the utilisation of blood. J Evolution Med Dent.

